# 1-[(2-Chloro-3-quinol­yl)meth­yl]indoline-2,3-dione

**DOI:** 10.1107/S1600536810013966

**Published:** 2010-04-24

**Authors:** F. Nawaz Khan, S Mohana Roopan, Sriramakrishnaswamy Kone, Venkatesha R. Hathwar, M. Khawar Rauf

**Affiliations:** a Organic Chemistry Division, School of Science, VIT University, Vellore 632 014, Tamil Nadu, India; b Solid State and Structural Chemistry Unit, Indian Institute of Science, Bangalore 560 012, Karnataka, India; c Department of Chemistry, Quaid-i-Azam University, Islamabad 45320, Pakistan

## Abstract

In the title compound, C_18_H_11_ClN_2_O_2_, the isatin and 2-chloro-3-methyl­quinoline units are both almost planar, with r.m.s. deviations of 0.0075 and 0.0086 Å, respectively, and the dihedral angle between the mean planes of the two units is 83.13 (7)°. In the crystal, a weak inter­molecular C—H⋯ O inter­action links the mol­ecules into chains along the *c* axis.

## Related literature

For background to the use of *N*-substituted indole-2,3-diones as inter­mediates and synthetic precursors for the preparation of heterocyclic compounds, see: Silaicheva *et al.* (2009 ▶). For the biological activity of *N*-substituted indole-2,3-diones, see: Vine *et al.* (2007 ▶). For reference bond lengths, see: Allen *et al.* (1987 ▶).
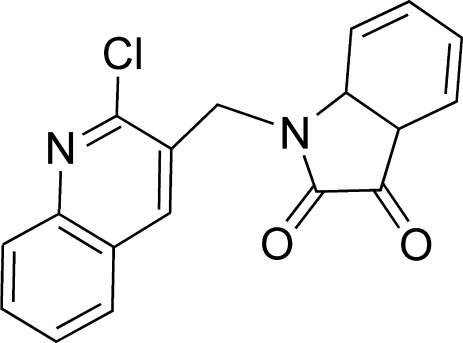


## Experimental

### 

#### Crystal data

C_18_H_11_ClN_2_O_2_
*M* *_r_* = 322.74
Monoclinic, 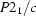

*a* = 21.4984 (8) Å
*b* = 5.3061 (2) Å
*c* = 13.0356 (4) Å
β = 99.718 (3)°
*V* = 1465.67 (9) Å^3^
*Z* = 4
Mo *K*α radiation
μ = 0.27 mm^−1^
*T* = 293 K
0.33 × 0.30 × 0.27 mm

#### Data collection

Oxford Diffraction Excalibur diffractometer
17920 measured reflections
2724 independent reflections
1731 reflections with *I* > 2σ(*I*)
*R*_int_ = 0.050

#### Refinement

*R*[*F* ^2^ > 2σ(*F* ^2^)] = 0.037
*wR*(*F* ^2^) = 0.085
*S* = 0.90
2724 reflections
208 parameters
H-atom parameters constrained
Δρ_max_ = 0.21 e Å^−3^
Δρ_min_ = −0.23 e Å^−3^

### 

Data collection: *CrysAlis PRO* (Oxford Diffraction, 2009 ▶); cell refinement: *CrysAlis PRO*; data reduction: *CrysAlis PRO*; program(s) used to solve structure: *SHELXS97* (Sheldrick, 2008 ▶); program(s) used to refine structure: *SHELXL97* (Sheldrick, 2008 ▶); molecular graphics: *ORTEP-3 for Windows* (Farrugia, 1997 ▶); software used to prepare material for publication: *WinGX* (Farrugia, 1999 ▶).

## Supplementary Material

Crystal structure: contains datablocks I, global. DOI: 10.1107/S1600536810013966/pv2276sup1.cif

Structure factors: contains datablocks I. DOI: 10.1107/S1600536810013966/pv2276Isup2.hkl

Additional supplementary materials:  crystallographic information; 3D view; checkCIF report

## Figures and Tables

**Table 1:** Hydrogen-bond geometry (Å, °)

*D*—H⋯*A*	*D*—H	H⋯*A*	*D*⋯*A*	*D*—H⋯*A*
C17—H17⋯O2^i^	0.93	2.48	3.367 (3)	160

Symmetry code: (i) 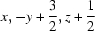.

**Fig. 1.**
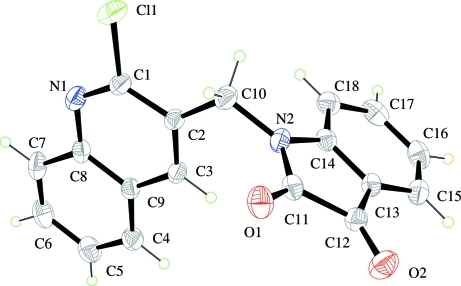
Molecular structure of the title compound showing atom numbering scheme. Displacement ellipsoids are drawn at the 30% probability level.

**Fig. 2.**
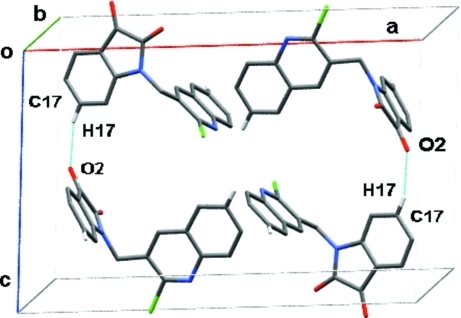
Perspective view of the molecular packing of the title compound in the unit cell down the b-axis.

## supplementary crystallographic information

### Comment

N-substituted indole-2,3-diones have been frequently used as intermediates and synthetic precursors for the preparation of a wide variety of heterocyclic compounds (Silaicheva *et al.*, 2009). In addition, they possess different biological activities such as cytotoxicity, antiviral activity and selective caspase inhibitions, etc. (Vine *et al.*, 2007). We have synthesized a novel isatin derivative and determined its crystal structure which is presented in this article.

In the title  molecule, the atoms (C11—C18/N2/O1/O2) of the isatin moiety and 2-chloro-3-methylquinoline group (C1—C8/N1/Cl1) are individually planar with maximum r.m.s. deviations of 0.0075 and 0.0086 Å, respectively, from their mean-planes. The dihedral angle between the two ring systems is 83.13 (7)°. The bond distances and angles in the title compound are as expected (Allen *et al.*, 1987). There is a weak intermolecular interaction C17—H17··· O2 linking the molecules into chains along the *c*-axis.

### Experimental

2-Chloro-3-chloromethylquinoline (210 mg, 1 mmol), KO^t^Bu (112 mg, 1 mmol) and isatin (147 mg, 1 mmol) in tetrahydrofuran (10 ml) were taken in a round bottemed flask and the mixture was refluxed at 70 W for 3 min. Ethylacetate (30 ml) was poured into the reaction mixture and filtered off. The filtrate was subjected to column chromatography packed with silica and ethyl acetate/petroleum ether was used as the eluant (4:1). Crystals of suitable quality were grown by slow evaporation from a solution of the title compound in dichloromethane.

### Refinement

Hydrogen atoms were placed in calculated positions at C—H = 0.93 and 0.97 Å, for aryl and methylene type H atoms, respectively, and were included in the refinement in riding model approximation, with U_iso_(H) set to 1.2U_eq_(C).

### Crystal data

**Table:**

C_18_H_11_ClN_2_O_2_	*F*(000) = 664
*M**_r_* = 322.74	*D*_x_ = 1.463 Mg m^−^^3^
Monoclinic, *P*2_1_/*c*	Mo *K*α radiation, λ = 0.71073 Å
Hall symbol: -P 2ybc	Cell parameters from 2724 reflections
*a* = 21.4984 (8) Å	θ = 2.9–25.5°
*b* = 5.3061 (2) Å	µ = 0.27 mm^−^^1^
*c* = 13.0356 (4) Å	*T* = 293 K
β = 99.718 (3)°	Block, orange
*V* = 1465.67 (9) Å^3^	0.33 × 0.30 × 0.27 mm
*Z* = 4	

### Data collection

**Table:**

Oxford Diffraction Excalibur diffractometer	1731 reflections with *I* > 2σ(*I*)
Radiation source: fine-focus sealed tube	*R*_int_ = 0.050
graphite	θ_max_ = 25.5°, θ_min_ = 2.9°
Detector resolution: 0 pixels mm^-1^	*h* = −26→26
ω scans	*k* = −6→6
17920 measured reflections	*l* = −15→15
2724 independent reflections	

### Refinement

**Table:**

Refinement on *F*^2^	Primary atom site location: structure-invariant direct methods
Least-squares matrix: full	Secondary atom site location: difference Fourier map
*R*[*F*^2^ > 2σ(*F*^2^)] = 0.037	Hydrogen site location: inferred from neighbouring sites
*wR*(*F*^2^) = 0.085	H-atom parameters constrained
*S* = 0.90	*w* = 1/[σ^2^(*F*_o_^2^) + (0.0447*P*)^2^] where *P* = (*F*_o_^2^ + 2*F*_c_^2^)/3
2724 reflections	(Δ/σ)_max_ < 0.001
208 parameters	Δρ_max_ = 0.21 e Å^−^^3^
0 restraints	Δρ_min_ = −0.23 e Å^−^^3^

### Special details

**Table:**

Geometry. All esds (except the esd in the dihedral angle between two l.s. planes) are estimated using the full covariance matrix. The cell esds are taken into account individually in the estimation of esds in distances, angles and torsion angles; correlations between esds in cell parameters are only used when they are defined by crystal symmetry. An approximate (isotropic) treatment of cell esds is used for estimating esds involving l.s. planes.
Refinement. Refinement of *F*^2^ against ALL reflections. The weighted *R*-factor wR and goodness of fit *S* are based on *F*^2^, conventional *R*-factors *R* are based on *F*, with *F* set to zero for negative *F*^2^. The threshold expression of *F*^2^ > σ(*F*^2^) is used only for calculating *R*-factors(gt) etc. and is not relevant to the choice of reflections for refinement. *R*-factors based on *F*^2^ are statistically about twice as large as those based on *F*, and *R*- factors based on ALL data will be even larger.

### Fractional atomic coordinates and isotropic or equivalent isotropic displacement parameters (Å^2^)

**Table:**

	*x*	*y*	*z*	*U*_iso_*/*U*_eq_	
Cl1	0.32510 (3)	1.40527 (10)	0.51979 (4)	0.0614 (2)	
N1	0.38948 (7)	1.0493 (3)	0.45048 (11)	0.0458 (4)	
C2	0.28707 (8)	1.1294 (3)	0.34507 (13)	0.0360 (4)	
C3	0.29797 (8)	0.9460 (3)	0.27719 (13)	0.0379 (4)	
H3	0.2675	0.9121	0.2192	0.046*	
N2	0.18214 (7)	1.2157 (3)	0.24049 (11)	0.0398 (4)	
C13	0.10081 (8)	1.0002 (3)	0.13973 (13)	0.0376 (4)	
C1	0.33623 (9)	1.1692 (3)	0.43088 (13)	0.0406 (5)	
C14	0.13853 (8)	1.0147 (3)	0.23746 (14)	0.0377 (4)	
C8	0.39988 (8)	0.8632 (3)	0.38138 (14)	0.0407 (5)	
O1	0.20473 (6)	1.5156 (3)	0.12348 (11)	0.0579 (4)	
C4	0.36754 (9)	0.6131 (3)	0.22547 (15)	0.0481 (5)	
H4	0.3382	0.5751	0.1666	0.058*	
O2	0.10171 (7)	1.2586 (3)	−0.01339 (11)	0.0635 (4)	
C10	0.22789 (9)	1.2877 (3)	0.33140 (14)	0.0463 (5)	
H10A	0.2085	1.2717	0.3931	0.056*	
H10B	0.2392	1.4632	0.3249	0.056*	
C9	0.35465 (8)	0.8066 (3)	0.29322 (13)	0.0375 (4)	
C15	0.05482 (9)	0.8170 (4)	0.11805 (15)	0.0477 (5)	
H15	0.0300	0.8064	0.0525	0.057*	
C12	0.12083 (8)	1.1985 (3)	0.07592 (15)	0.0423 (5)	
C7	0.45672 (9)	0.7252 (4)	0.39988 (16)	0.0547 (6)	
H7	0.4871	0.7616	0.4576	0.066*	
C5	0.42260 (10)	0.4820 (4)	0.24572 (18)	0.0587 (6)	
H5	0.4306	0.3541	0.2010	0.070*	
C11	0.17522 (9)	1.3370 (4)	0.14680 (15)	0.0418 (5)	
C6	0.46691 (10)	0.5389 (4)	0.33319 (19)	0.0588 (6)	
H6	0.5043	0.4475	0.3463	0.071*	
C18	0.13105 (9)	0.8492 (4)	0.31574 (15)	0.0476 (5)	
H18	0.1561	0.8586	0.3812	0.057*	
C16	0.04657 (9)	0.6503 (4)	0.19561 (18)	0.0542 (6)	
H16	0.0156	0.5264	0.1829	0.065*	
C17	0.08426 (10)	0.6670 (4)	0.29230 (18)	0.0552 (6)	
H17	0.0781	0.5524	0.3436	0.066*	

### Atomic displacement parameters (Å^2^)

**Table:**

	*U*^11^	*U*^22^	*U*^33^	*U*^12^	*U*^13^	*U*^23^
Cl1	0.0765 (4)	0.0609 (4)	0.0442 (3)	−0.0082 (3)	0.0028 (3)	−0.0161 (2)
N1	0.0427 (10)	0.0504 (10)	0.0406 (9)	−0.0072 (8)	−0.0039 (8)	0.0046 (8)
C2	0.0363 (10)	0.0362 (10)	0.0346 (10)	−0.0046 (9)	0.0038 (8)	−0.0006 (8)
C3	0.0337 (10)	0.0422 (11)	0.0359 (10)	−0.0036 (9)	0.0000 (8)	0.0003 (9)
N2	0.0341 (9)	0.0400 (9)	0.0425 (10)	0.0007 (7)	−0.0016 (7)	−0.0043 (7)
C13	0.0320 (10)	0.0364 (10)	0.0436 (12)	0.0019 (9)	0.0040 (9)	0.0000 (9)
C1	0.0460 (12)	0.0415 (11)	0.0335 (11)	−0.0096 (10)	0.0042 (9)	−0.0009 (9)
C14	0.0340 (11)	0.0335 (10)	0.0461 (12)	0.0066 (9)	0.0078 (9)	−0.0017 (9)
C8	0.0319 (11)	0.0430 (11)	0.0457 (12)	−0.0052 (9)	0.0027 (9)	0.0079 (9)
O1	0.0489 (9)	0.0526 (9)	0.0719 (10)	−0.0123 (7)	0.0092 (7)	0.0042 (7)
C4	0.0427 (12)	0.0496 (12)	0.0520 (13)	0.0032 (10)	0.0078 (10)	−0.0040 (10)
O2	0.0690 (10)	0.0721 (10)	0.0457 (9)	−0.0133 (8)	−0.0013 (8)	0.0077 (7)
C10	0.0439 (12)	0.0458 (11)	0.0465 (12)	0.0017 (10)	0.0004 (10)	−0.0106 (9)
C9	0.0326 (11)	0.0405 (10)	0.0388 (11)	−0.0039 (9)	0.0045 (9)	0.0038 (9)
C15	0.0394 (12)	0.0460 (11)	0.0554 (13)	−0.0005 (10)	0.0008 (10)	−0.0040 (10)
C12	0.0402 (12)	0.0448 (11)	0.0410 (12)	0.0026 (9)	0.0045 (10)	−0.0006 (10)
C7	0.0337 (12)	0.0623 (14)	0.0632 (14)	−0.0041 (11)	−0.0062 (10)	0.0136 (12)
C5	0.0492 (14)	0.0546 (13)	0.0741 (16)	0.0050 (11)	0.0160 (12)	−0.0028 (11)
C11	0.0330 (11)	0.0405 (11)	0.0516 (13)	0.0026 (10)	0.0069 (9)	0.0001 (10)
C6	0.0395 (13)	0.0548 (14)	0.0824 (17)	0.0074 (11)	0.0107 (12)	0.0099 (12)
C18	0.0476 (13)	0.0477 (12)	0.0477 (12)	0.0142 (11)	0.0087 (10)	0.0045 (10)
C16	0.0413 (13)	0.0421 (12)	0.0810 (17)	−0.0023 (10)	0.0155 (12)	−0.0007 (12)
C17	0.0576 (14)	0.0415 (12)	0.0726 (16)	0.0104 (11)	0.0282 (13)	0.0122 (11)

### Geometric parameters (Å, °)

**Table:**

Cl1—C1	1.7503 (18)	C4—C9	1.412 (2)
N1—C1	1.297 (2)	C4—H4	0.9300
N1—C8	1.380 (2)	O2—C12	1.210 (2)
C2—C3	1.362 (2)	C10—H10A	0.9700
C2—C1	1.419 (3)	C10—H10B	0.9700
C2—C10	1.510 (2)	C15—C16	1.377 (3)
C3—C9	1.411 (2)	C15—H15	0.9300
C3—H3	0.9300	C12—C11	1.548 (3)
N2—C11	1.366 (2)	C7—C6	1.358 (3)
N2—C14	1.416 (2)	C7—H7	0.9300
N2—C10	1.457 (2)	C5—C6	1.390 (3)
C13—C15	1.381 (2)	C5—H5	0.9300
C13—C14	1.392 (2)	C6—H6	0.9300
C13—C12	1.451 (3)	C18—C17	1.391 (3)
C14—C18	1.376 (2)	C18—H18	0.9300
C8—C9	1.407 (2)	C16—C17	1.381 (3)
C8—C7	1.410 (3)	C16—H16	0.9300
O1—C11	1.208 (2)	C17—H17	0.9300
C4—C5	1.360 (3)		
			
C1—N1—C8	117.22 (16)	C8—C9—C3	118.02 (17)
C3—C2—C1	115.56 (17)	C8—C9—C4	118.98 (17)
C3—C2—C10	123.70 (16)	C3—C9—C4	123.00 (17)
C1—C2—C10	120.73 (16)	C16—C15—C13	118.58 (18)
C2—C3—C9	121.17 (17)	C16—C15—H15	120.7
C2—C3—H3	119.4	C13—C15—H15	120.7
C9—C3—H3	119.4	O2—C12—C13	131.09 (18)
C11—N2—C14	110.98 (15)	O2—C12—C11	123.28 (17)
C11—N2—C10	124.07 (15)	C13—C12—C11	105.62 (16)
C14—N2—C10	124.94 (15)	C6—C7—C8	119.8 (2)
C15—C13—C14	120.82 (16)	C6—C7—H7	120.1
C15—C13—C12	131.64 (17)	C8—C7—H7	120.1
C14—C13—C12	107.54 (16)	C4—C5—C6	120.1 (2)
N1—C1—C2	126.69 (17)	C4—C5—H5	119.9
N1—C1—Cl1	115.82 (14)	C6—C5—H5	119.9
C2—C1—Cl1	117.49 (15)	O1—C11—N2	127.74 (18)
C18—C14—C13	121.36 (17)	O1—C11—C12	126.75 (18)
C18—C14—N2	128.29 (17)	N2—C11—C12	105.50 (16)
C13—C14—N2	110.35 (15)	C7—C6—C5	121.4 (2)
N1—C8—C9	121.33 (17)	C7—C6—H6	119.3
N1—C8—C7	119.42 (18)	C5—C6—H6	119.3
C9—C8—C7	119.24 (18)	C14—C18—C17	116.82 (19)
C5—C4—C9	120.46 (19)	C14—C18—H18	121.6
C5—C4—H4	119.8	C17—C18—H18	121.6
C9—C4—H4	119.8	C15—C16—C17	120.00 (19)
N2—C10—C2	112.95 (14)	C15—C16—H16	120.0
N2—C10—H10A	109.0	C17—C16—H16	120.0
C2—C10—H10A	109.0	C16—C17—C18	122.42 (19)
N2—C10—H10B	109.0	C16—C17—H17	118.8
C2—C10—H10B	109.0	C18—C17—H17	118.8
H10A—C10—H10B	107.8		
			
C1—C2—C3—C9	0.3 (2)	C2—C3—C9—C4	179.27 (16)
C10—C2—C3—C9	179.03 (16)	C5—C4—C9—C8	0.6 (3)
C8—N1—C1—C2	−0.1 (3)	C5—C4—C9—C3	−178.91 (17)
C8—N1—C1—Cl1	−179.53 (12)	C14—C13—C15—C16	0.7 (3)
C3—C2—C1—N1	−0.2 (3)	C12—C13—C15—C16	179.68 (17)
C10—C2—C1—N1	−178.90 (17)	C15—C13—C12—O2	1.3 (3)
C3—C2—C1—Cl1	179.28 (12)	C14—C13—C12—O2	−179.6 (2)
C10—C2—C1—Cl1	0.5 (2)	C15—C13—C12—C11	−179.36 (18)
C15—C13—C14—C18	−0.5 (3)	C14—C13—C12—C11	−0.28 (18)
C12—C13—C14—C18	−179.71 (16)	N1—C8—C7—C6	178.92 (17)
C15—C13—C14—N2	179.64 (15)	C9—C8—C7—C6	−0.5 (3)
C12—C13—C14—N2	0.45 (19)	C9—C4—C5—C6	−0.4 (3)
C11—N2—C14—C18	179.71 (17)	C14—N2—C11—O1	179.81 (17)
C10—N2—C14—C18	−1.1 (3)	C10—N2—C11—O1	0.6 (3)
C11—N2—C14—C13	−0.46 (19)	C14—N2—C11—C12	0.25 (18)
C10—N2—C14—C13	178.72 (15)	C10—N2—C11—C12	−178.93 (15)
C1—N1—C8—C9	0.2 (3)	O2—C12—C11—O1	−0.2 (3)
C1—N1—C8—C7	−179.23 (16)	C13—C12—C11—O1	−179.55 (17)
C11—N2—C10—C2	−98.40 (18)	O2—C12—C11—N2	179.41 (17)
C14—N2—C10—C2	82.5 (2)	C13—C12—C11—N2	0.02 (18)
C3—C2—C10—N2	2.8 (2)	C8—C7—C6—C5	0.7 (3)
C1—C2—C10—N2	−178.58 (16)	C4—C5—C6—C7	−0.2 (3)
N1—C8—C9—C3	0.0 (3)	C13—C14—C18—C17	0.2 (3)
C7—C8—C9—C3	179.40 (16)	N2—C14—C18—C17	180.00 (16)
N1—C8—C9—C4	−179.55 (15)	C13—C15—C16—C17	−0.6 (3)
C7—C8—C9—C4	−0.2 (3)	C15—C16—C17—C18	0.3 (3)
C2—C3—C9—C8	−0.3 (2)	C14—C18—C17—C16	−0.1 (3)

### Hydrogen-bond geometry (Å, °)

**Table:**

*D*—H···*A*	*D*—H	H···*A*	*D*···*A*	*D*—H···*A*
C3—H3···N2	0.93	2.49	2.842 (2)	102
C17—H17···O2^i^	0.93	2.48	3.367 (3)	160

Symmetry codes: (i) *x*, −*y*+3/2, *z*+1/2.
